# Development and Characterization of Wheat-*Thinopyrum elongatum* 1B-1E Translocation Lines with Fusarium Head Blight Resistance

**DOI:** 10.3390/plants14243805

**Published:** 2025-12-13

**Authors:** Can Wang, Zixuan Liu, Xingwen Wang, Xiaoni Wang, Xinyue Guo, Haitong He, Aiping Wang, Yaping Cao, Wei Zhang

**Affiliations:** 1 Shanxi Hou Ji Laboratory, College of Agriculture, Shanxi Agricultural University, Jinzhong 030801, China; 2 Wheat Research Institute, Shanxi Agricultural University, Linfen 041000, China

**Keywords:** wheat, FHB resistance, gene introgression, molecular mapping

## Abstract

Fusarium head blight (FHB) is a devastating disease of wheat (*Triticum aestivum*) globally. Utilizing resistance genes from wild relatives like *Thinopyrum elongatum* offers a promising approach for genetic improvement. We introgressed FHB resistance from *Th. elongatum* chromosome 1E into common wheat by inducing homoeologous recombination with wheat chromosome 1B using the *ph1b* mutant. From a population of 376 BC_1_F_2_ individuals, we identified 19 independent 1E-1B recombinant lines using KASP markers and fluorescence genomic in situ hybridization (FGISH). High-resolution genotyping with a 130K SNP array precisely mapped recombination breakpoints, revealing a hotspot in the distal long arm. Further phenotypic evaluation revealed that 11 recombinants exhibited significantly enhanced FHB resistance compared to the susceptible Chinese Spring (CS) control. Cytogenetic and physical mapping localized the resistance to a ~48 Mb subtelomeric interval on the long arm of chromosome 1E. This study provides novel wheat germplasm with improved FHB resistance, delineates the physical location of the resistance gene(s) on chromosome 1E, and demonstrates an efficient strategy for precise introgression of valuable genes from wild relatives into cultivated wheat.

## 1. Introduction

Fusarium head blight (FHB), a devastating fungal disease of wheat (*Triticum aestivum*) primarily caused by *Fusarium graminearum*, poses a severe threat to global wheat production. Beyond significant yield losses, FHB contaminates grain with harmful mycotoxins, such as deoxynivalenol (DON), compromising food and feed safety [1,2,3]. The disease is notoriously difficult to control due to the broad host range of the pathogen and its dependence on unpredictable climatic conditions for infection [4,5]. Consequently, the development of genetically resistant cultivars is widely considered the most sustainable and effective strategy for FHB management [6,7].

Modern wheat breeding, while successful in boosting yield, has led to a narrow genetic base, limiting the availability of FHB resistance sources within the primary wheat gene pool [8,9,10]. Wild relatives of wheat represent a critical reservoir of untapped genetic diversity, harboring valuable genes for resistance to biotic and abiotic stresses [11,12,13]. Indeed, several of the few formally designated FHB resistance genes, including *Fhb3*, *Fhb6*, and *Fhb7*, originate from wild species [14,15,16]. A major bottleneck in utilizing these genes is the stringent regulation of meiotic recombination between wheat and its wild relatives, which prevents the direct introgression of desirable chromosomal segments.

The pairing homoeologous (*Ph*) gene acts as a guardian of genome stability by enforcing strict homologous chromosome pairing during meiosis, thereby preventing recombination between genetically similar but evolutionarily divergent homoeologous chromosomes. [17,18,19,20]. The discovery of the *ph1b* mutant, which lacks *Ph1* function, has been transformative for wheat chromosome engineering [19,21]. By disabling this regulatory checkpoint, the *ph1b* mutation enables controlled homoeologous recombination between wheat chromosomes and those of its wild relatives, such as *Thinopyrum*, *Aegilops* and *Dasypyrum* species [11,22,23,24,25,26]. This breakthrough allows precise introgression of alien chromosomal segments into the wheat genome, circumventing traditional hybridization barriers and accelerating the development of novel germplasm [27,28,29,30,31].

Advances in cytogenetic and genomic technologies have further accelerated this progress. Genomic in situ hybridization (GISH) and fluorescent *in situ* hybridization (FISH) allow for the precise visual identification of introgressed segments [32,33,34,35,36]. Coupled with high-throughput genotyping platforms, such as SNP arrays [37,38,39,40,41], and the availability of complete wheat reference genomes [42,43,44,45,46], researchers can now physically map recombination breakpoints with high resolution, characterize introgressed segments, and partition wheat chromosomes into defined physical intervals [32,35,47,48,49].

As a wild relative of common wheat, *Thinopyrum elongatum* (2n = 2x = 14) serves as a valuable genetic reservoir for disease resistance. Previous studies using wheat–*Th. elongatum* cytogenetic lines have localized resistance factors on chromosomes 1E and 7E [50,51,52], with the gene on 7E (*Fhb7*) having been successfully introgressed into wheat from either diploid *Th. elongatum* or decaploid *Th. ponticum* via engineered translocations [30,36,52,53,54,55,56]. In contrast, the resistance on chromosome 1E remains less characterized and underutilized.

Here, we report the engineering of *Th. elongatum* chromosome 1E through *ph1b*-induced homoeologous recombination with wheat chromosome 1B to introgress FHB resistance into the wheat genome. We physically characterized the newly developed 1E-1B recombinant lines using FISH/GISH and genotyped them with a high-throughput 130K SNP array. This integrated approach allowed us to map the FHB resistance to a sub-distal region of the 1EL arm. Our work not only provides a unique physical framework for chromosomes 1B and 1E but also establishes a scalable strategy for unlocking the genetic potential of wheat’s wild relatives to address pressing agricultural challenges.

## 2. Results

### 2.1. Construction of ph1b-Induced 1E/1B Homoeologous Recombination Population

**Figure 1:**
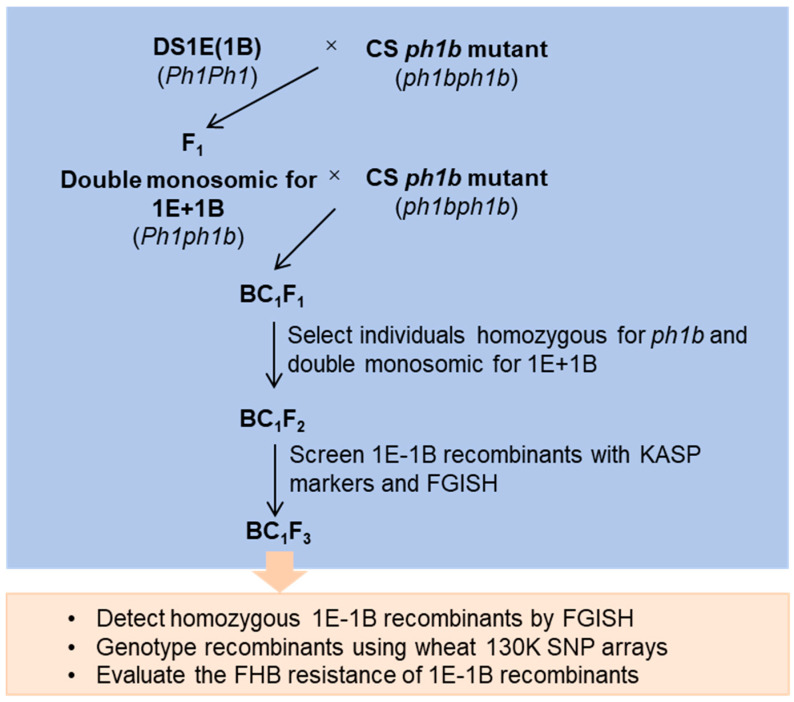
Diagram showing strategy for induction, detection and characterization of 1E-1B homoeologous recombinants.

**Figure 2:**
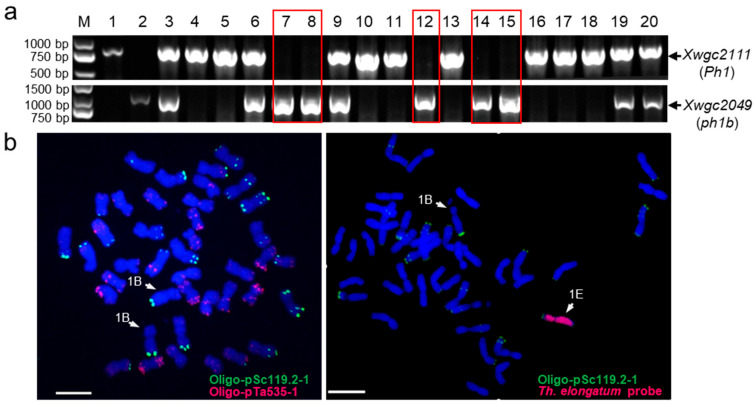
Selection of individuals homozygous for *ph1b* and double monosomic for 1E + 1B from BC_1_F_1_ population. (**a**) Genotypes of Xwgc2111 (*Ph1*) and Xwgc2049 (*ph1b*) of each line from the BC_1_F_1_ population. M: DNA marker; 1: CS; 2: CS ph1b; 3–20: individuals from 1E-1B population. The highlighted lane 7, 8, 12, 14 and 15 indicated the genotypes homozygous for *ph1b* allele. (**b**) FGISH verification of lines double monosomic for 1E + 1B (**right**) and the CS as control (**left**). The E genome probe was labeled with digoxigenin (DIG) and visualized as a red fluorescent signal using an anti-digoxigenin-rhodamine conjugate. Bar = 10 μm.

To induce homoeologous recombination, the initial plants DS 1E(1B) were crossed and subsequently backcrossed to the CS *ph1b* mutant to generate BC_1_F_1_ populations (Figure 1). We genotyped 18 BC_1_F_1_ individuals using the *ph1b*-specific markers *Xwgc2111* and *Xwgc2049* and identified five homozygous mutants (Figure 2a). These five lines were then screened for 1E + 1B double-monosomics using FGISH. Chromosome 1E was distinguished using a *Th. elongatum* genomic DNA probe, while chromosome 1B was identified by its prominent short-arm satellite and the Oligo-pSc119.2-1 signal on its long arm (Figure 2b). This screen identified a single 1E + 1B double-monosomic line in the *ph1b* mutant background. This plant was self-pollinated to induce homoeologous recombination between the *Th. elongatum* 1E and wheat 1B chromosomes, generating a BC_1_F_2_ population of 405 progenies for screening 1E-1B recombinants.

### 2.2. Screening and Verification of 1E-1B Recombinants by KASP Markers and GISH

**Figure 3:**
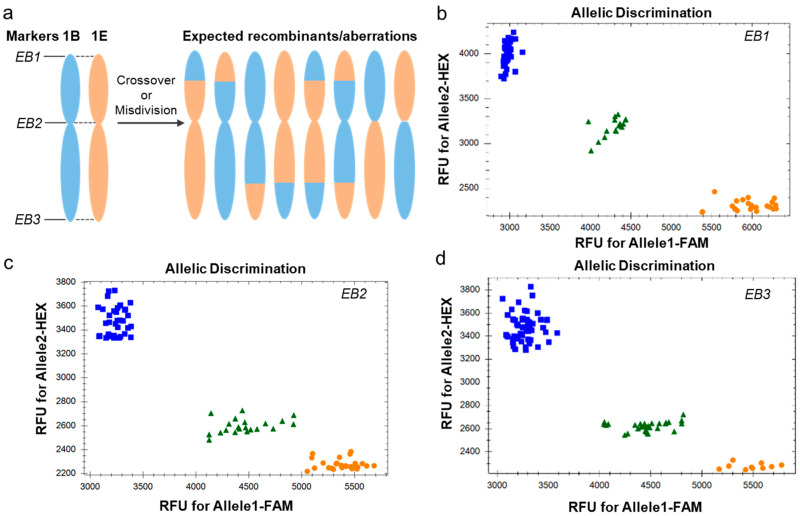
Application of KASP markers in detection of 1E-1B recombinants. (**a**) Chromosome ideogram showing the expected 1E-1B translocations by KASP screening. (**b**–**d**) allelic discrimination plots showing the genotypes of BC_1_F_2_ individuals applied with KASP markers EB1, EB2, and EB3, respectively. Orange circles: Homozygous for allele 1; Blue squares: Homozygous for allele 2; Green triangles: Heterozygous (allele 1/allele 2). RFU: relative fluorescence unit.

SNPs distinguishing the homoeologous chromosomes 1E and 1B were identified by aligning the *Th. elongatum* 1E sequence (genome assembly: ASM1179987v1) to the CS reference genome (IWGSC RefSeq v2.1) by BLAST online. Ideal candidates for diagnostic marker development were SNPs polymorphic between 1B and 1E, while being monomorphic within the other wheat subgenomes (e.g., 1D) to ensure specificity. Eventually, we validated three KASP markers (*EB1*, *EB2*, and *EB3*) as codominant markers for discriminating between wheat chromosome 1B and *Th. elongatum* 1E (Table 1). These markers, which are distributed across pericentromeric and distal regions of the short and long arms, were deployed to screen the population for 1E-1B recombinant events (Figure 3).

**Figure 4:**
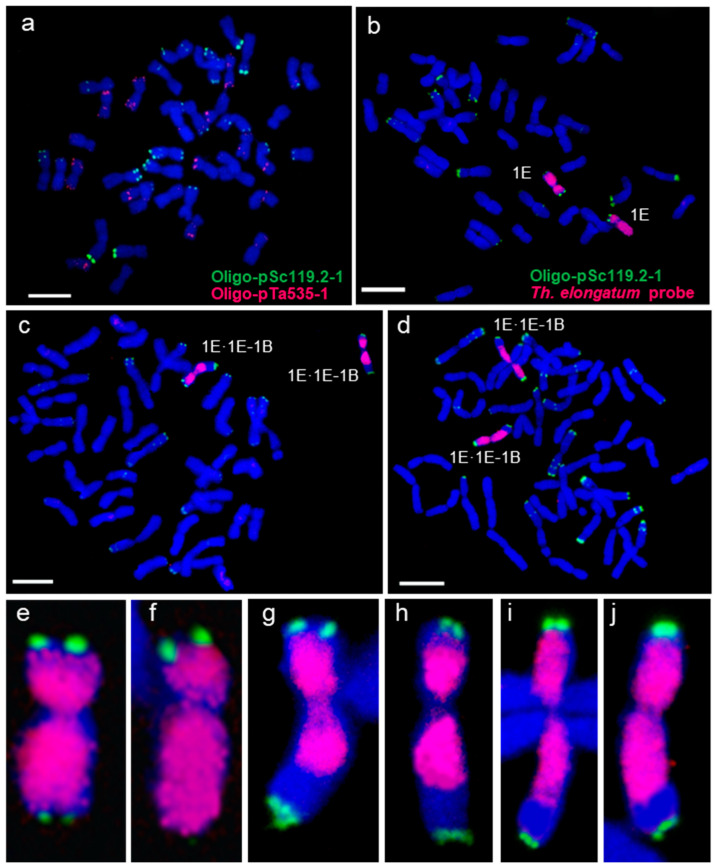
Identification of 1E-1B homozygous recombinants by FGISH. (**a**) CS painted with Oligo-pSc119.2-1 and Oligo-pTa535-1; (**b**–**d**) Oligo-pSc119.2-1 and *Th. elongatum* probes painted DS 1E (1B) and 1E·1E-1B homozygous recombinants; (**e**,**f**) chromosomes 1E cropped from (**b**); (**g**,**h**) chromosomes 1E·1E-1B cropped from (**c**); (**i**,**j**) translocated chromosomes 1E·1E-1B cropped from (**d**). Bar = 10 μm.

From a population of 405 sown BC_1_F_2_ seeds, 376 germinated and were screened with KASP markers, revealing 30 potential recombinants (Supplementary S1). Subsequent validation by FGISH confirmed 19 true recombinants, yielding a recombination frequency of 5.05% (Supplementary S2). All confirmed events occurred on the long arm, with no evidence of misdivision products such as Robertsonian translocations or telocentric chromosomes. All initially recovered recombinants were heterozygous, carrying a single 1E-1B recombinant chromosome. Homozygous lines were subsequently selected from the self-pollinated progenies of validated recombinants using FGISH (Figure 4). To date, we have obtained one to four homozygous lines for each of the 1E-1B recombinants (Supplementary S3).

### 2.3. Homoeologous Recombination-Based Delineation and Physical Mapping of Wheat Chromosome 1B

**Figure 5:**
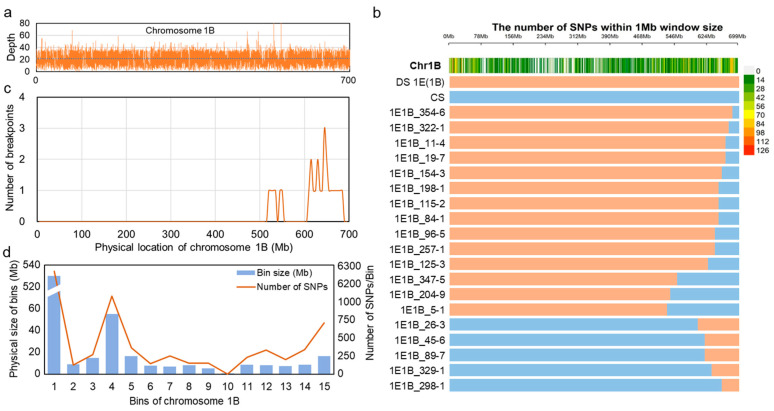
Characterization of 1E-1B recombinants with 130K SNPs array. (**a**) Sequencing depth of target loci on chromosomes 1A and 1B. (**b**) The SNPs and distribution on translocated 1E-1B chromosomes. Blue bars represent chromosome 1B segments and brown bars for 1E segments. (**c**) Scanning for breakpoints on translocated 1E-1B chromosomes in 10-Mb sliding windows with 5-Mb steps. (**d**) Composite bin map of wheat chromosome 1B. Vertical bars indicate physical size of the bins, and curved lines indicate SNP numbers within each bin.

The identified homozygous recombinants and the parental lines (CS and DS 1E(1B)) were genotyped using the 130 K wheat SNP array with an average sequencing depth of 20× (Figure 5a and Supplementary S4). To distinguish 1E from 1B chromatin, we selected target SNPs with unambiguous physical positions on chromosome 1B. SNPs genotyped as “0” (reference allele) were assigned to 1B, while those genotyped as “1” (alternative allele) or “NULL” were assigned to 1E. Using these criteria, a total of 10,443 SNPs polymorphic between 1E and 1B were selected to characterize the recombinant lines (Figure 5b and Supplementary S5). Analysis of 19 1E-1B recombinants revealed the recombination breakpoints were predominantly located on a 523.0–682.3 Mb interval within the distal long arm (Figure 5c).

Based on the SNP data from 19 recombinant lines, chromosome 1B was partitioned into 15 bins, to which 10,443 SNPs were assigned. The physical size of these bins ranged from 0.4 to 570.8 Mb, containing between 6 and 6251 SNPs each (Figure 5d and Supplementary S5). The chromosomal bins in the proximal and short arm regions were significantly larger than those in the distal long arm, indicating lower frequency of meiotic homoeologous recombination in the peri-centromeric and short arm regions compared to the distal regions of the long arm of 1E-1B homoeologous pair.

### 2.4. Evaluation of the Reaction of 1E-1B Recombinants to FHB

**Figure 6:**
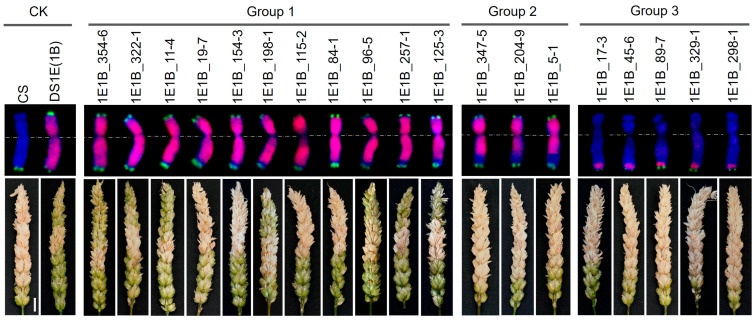
FGISH patterns of 1B, 1E and 1E-1B translocated chromosomes (**top**) and the reactions of CS, DS 1E(1B), and 1E-1B translocation lines to FHB infection (**bottom**). *Th. elongatum* genome was labeled in red as GISH probe; Oligo-pSc119.2-1 (green) was labeled as FISH probes; wheat chromatin was painted as blue by DAPI.

The homozygous 1B-1E recombinants and parental lines (CS and DS 1E(1B)) were evaluated for FHB resistance by the single floret inoculation in two greenhouse seasons. We observed that in the CS line, most spikes were bleached, with lesions expanding across nearly the entire spike. In contrast, only a small proportion of spikes in the DS 1E(1B) lines showed bleaching, and lesions were typically confined to a few spikelets (Figure 6). Statistical analysis confirmed that the DS 1E(1B) lines exhibited significantly lower FHB severity than CS in both the 2024 and 2025 growing seasons. Although the DS 1E(1B) lines showed slightly reduced resistance in 2025, their FHB severity remained statistically significantly lower than that of CS. These results indicate that chromosome 1E carries gene(s) conferring FHB resistance (Table 2).

Upon comparison with CS and DS 1E(1B), 11 of the 19 recombinants demonstrated enhanced FHB resistance, showing significantly reduced disease severity across both growing seasons. The remaining eight recombinants exhibited susceptibility levels comparable to or greater than CS. (Table 2). Based on integrated FHB phenotyping and cytogenetic analysis, we categorized the recombinants into three distinct groups (Figure 6). Group 1 recombinants carry a large segment of 1E chromatin spanning the entire short arm and most of the long arm. All lines in this group displayed significantly lower FHB severity than CS in both seasons. Among them, five lines (1E1B_354-6, 1E1B_322-1, 1E1B_11-4, 1E1B_257-1, 1E1B_125-3) conferred a resistance level comparable to the resistant parent DS 1E(1B), while the other six lines, despite a slight reduction in resistance, still maintained statistically significant resistance in the 2024 season. Group 2 recombinants, which carry a slightly shorter 1E chromatin segment, showed FHB severity levels similar to the susceptible CS control. Group 3 recombinants, carrying only a small distal segment of the 1E long arm, were clearly susceptible to FHB (Figure 6 and Table 2). These results indicate that FHB resistance gene(s) resides in the subtelomeric region of 1EL.

### 2.5. Molecular Mapping of the FHB Resistance Gene(s) on Th. elongatum 1E

**Figure 7:**
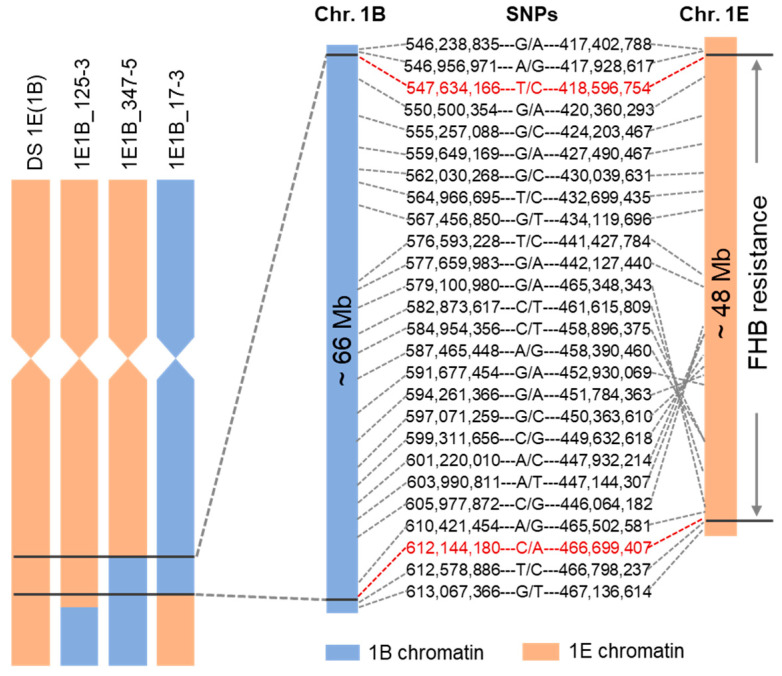
Physical mapping of FHB resistance gene(s) on chromosome 1E. DS 1E(1B): parental FHB resistant line; 1E-1B_125-3: recombinant resistant to FHB; 1E-1B_347-5 and 1E-1B_17-3: recombinants susceptible to FHB. Blue bars represent chromosome 1B segments and brown bars for 1E segments.

To further determine the size and the physical interval of the alien segments containing FHB resistance gene, we compared the architecture of translocated chromosomes with physical map derived by 130 K SNPs genotyping. The SNP-based physical maps of the recombinant chromosomes were highly consistent with their corresponding FGISH patterns in both the size and position of introgressed segments. The subtelomeric region of 1E, putatively containing FHB resistance gene(s), is homoeologous to the bin No.4 of chromosome 1B according to the SNPs genotypes (Figure 7). The Bin No. 4 on chromosome 1B is delimited by two recombination breakpoints. The proximal end is defined by the interval between SNPs 546,957,040 bp and 547,634,166 bp, and the distal end by the interval between SNPs 612,144,180 bp and 612,578,793 bp, yielding an estimated physical interval of ~66 Mb (Supplementary S4). We performed a BLASTn homology search of the contextual sequences spanning bin No. 4 on chromosome 1B against the *Th. elongatum* reference genome (ASM1179987v1) on NCBI. This analysis identified the homoeologous 1E segment, which is flanked by SNPs at positions 418,596,754 and 466,699,407 and has an estimated size of ~48 Mb (Figure 7). Since all FHB-resistant recombinants carry this ~48 Mb 1E segment, while all susceptible lines lack it, collectively indicates that this region constitutes the candidate interval harboring the FHB resistance gene(s).

## 3. Discussion

As a wild relative of wheat, *Th. elongatum* is regarded as a potential source of novel genes for wheat improvement, particularly for enhancing resistance to FHB [57]. To date, two FHB resistance loci have been identified on chromosomes 1E and 7E of *Th. elongatum*. The FHB resistance gene *Fhb7* on chromosome 7E has been extensively characterized and successfully introduced into commercial wheat cultivars through chromosome engineering [16,36,56]. In contrast, the resistance gene on chromosome 1E has not been thoroughly investigated, largely due to its comparatively weaker effect than that of *Fhb7*. Multiple 1E chromosome addition lines derived from diverse donor sources consistently exhibit varying degrees of enhanced FHB resistance compared to controls [50,51]. However, substitution lines show either increased or decreased resistance, suggesting that while chromosome 1E does carry FHB resistance gene(s), their expression may be influenced by the genotypic background of different recipient materials [51,58].

In the present study, the DS 1E(1B) substitution line displayed FHB infection rates of 25.8% and 42.8% across two growing seasons, significantly lower than the 70.0% and 78.0% observed in the CS control. In addition, the derived 1E-1B recombinants (categorized as group 1) carrying a large segment of *Th. elongatum* segment, exhibited reduced degree of FHB severity by comparing with CS, demonstrates that chromosome 1E carries effective FHB resistance gene(s) in the CS genetic background. While resistance is classically divided into five types (I–V) [59,60,61], this study was confined to an evaluation of Type II (resistance to within-spike spread) using single-floret inoculation. Key types such as resistance to mycotoxin accumulation (Type III), an important wheat resistance index measured by toxins like DON, were not examined. Therefore, subsequent work is necessary to investigate whether the observed resistance also confers reduced toxin accumulation in the recombinants.

The genomic affinity between wheat and its wild relatives enables the transfer of beneficial genes through meiotic homoeologous recombination, facilitating the development of compensating recombinant lines for alien gene introgression [62]. In this study, we employed a strategy to induce homoeologous recombination between *Th. elongatum* chromosome 1E and wheat chromosome 1B in BC_1_F_1_ individuals under the *ph1b* mutant background, followed by recovery of 1E-1B recombinant lines from the BC_1_F_2_ population. Previous studies report that the meiotic pairing rate between CS chromosome 1B and *Th. elongatum* 1E reaches approximately 16% in the *ph1b* background, whereas no pairing occurs in the presence of the functional *Ph1* allele [63]. Therefore, homologous recombination between 1E and 1B is expected to occur predominantly in the *ph1b* genetic context.

The recombined 1E-1B chromosomes are the products of a homoeologous recombination event, constituting a genetically compensatory transfer. Consequently, such recombinants typically demonstrate greater genetic stability than substitution lines. Furthermore, substitution lines are frequently compromised by linkage drag—the co-transfer of undesirable wild traits via non-target genes. Translocations with reduced alien chromatin help eliminate this linkage drag, thereby offering a more advantageous strategy for breeding applications. Unfortunately, in this study, all resistant lines carried a large *Th. elongatum* segment spanning the entire short arm and most of the long arm; no lines carrying only a small 1E segment on the long arm were identified. Therefore, the identified FHB-resistant translocations are best utilized as bridge materials for developing new, optimized recombinants with reduced alien chromatin and minimal linkage drag.

Although the recovery and detection of meiotic recombinants have traditionally been labor-intensive, recent advances in genomic technologies have substantially improved the efficiency of chromosome engineering [48,62]. In this study, we implemented an integrated strategy combining genomic and cytogenetic approaches. We first pre-screened large populations using SNP-based KASP markers diagnostic for the centromeric and terminal regions of chromosomes 1B and 1E, followed by verification with FGISH analysis. We totally identified 19 1E-1B recombinants from 376 individuals with a frequency of 5.05%. This approach streamlined the recombinant recovery process, making it more efficient than relying on cytogenetics alone. However, the observed homoeologous recombination frequency between chromosomes 1E and 1B is likely an underestimate due to limitations in the KASP marker system. First, the distal KASP markers *EB1* and *EB3* are located at positions 1,518,466 bp and 697,751,963 bp, respectively, on chromosome 1B (IWGSC RefSeq v2.1), which has a total length of 700,547,350 bp. Consequently, any recombination events occurring outside this ~1.52–697.75 Mb genomic interval would remain undetected. Second, the resolution of FGISH may be insufficient to detect recombination involving very small chromatin segments between 1E and 1B. Third, the marker system used cannot identify reciprocal recombinants in the BC_1_F_2_ population, further contributing to an underestimation of recombination frequency

The *ph1b* mutation induces homoeologous recombination not only between chromosomes 1B and 1E, but also among other group-1 chromosomes (e.g., 1A–1B and 1A–1D). This can lead to false positives in KASP marker analysis. Thus, all KASP-selected recombinants were further validated by FGISH. To distinguish between *Th. elongatum* and wheat chromatin in the translocated chromosomes, FGISH was performed using labeled *Th. elongatum* genomic DNA and the pSc119.2-1 oligonucleotide as probes. Chromosome 1E was characterized by strong hybridization signals from the *Th. elongatum* genomic DNA probe, accompanied by weak pSc119.2-1 signals at the terminal end of the long arm. In contrast, chromosome 1B was identified by the presence of a satellite on the short arm and strong pSc119.2-1 signals on the terminal end of long arm, which clearly distinguish it from homoeologous chromosomes 1A and 1D. Based on these cytogenetic markers, the 1B–1E recombinants were reliably identified in our FGISH analysis through a combination of satellite morphology, pSc119.2-1 signals, and the *Th. elongatum* probe. Only the recombinants show consistence evidence on KASP screening and FGISH analysis have been considered as 1E-1B recombinants with the false positives involving 1A/1D ruled out from substantial analysis.

By integrating 130K SNPs genotyping data with FGISH karyotyping, we constructed a composite bin map for wheat chromosome 1B. However, a high frequency of null alleles in the introgressed 1E segments precluded the development of a comparable map for *Th. elongatum* chromosome 1E, as the 130K SNP array was designed based on the wheat genome sequence. Despite this limitation, the recombination events and genotyping data enabled us to delineate chromosome 1E into well-defined physical bins. By performing BLASTn alignment of the SNP-flanking sequences against the *Th. elongatum* genome, we localized the FHB resistance locus to an approximately 48 Mb interval on chromosome 1E. As FHB resistance is typically quantitative, the number of candidate genes underlying this resistance remains unclear. Fine mapping the resistance gene(s) within this region will require a higher-resolution physical map.

We also identified a major recombination hotspot in the distal region of the long arm (∼570.8–682.3 Mb) of the 1E–1B homoeologs. Similar distal recombination hotspots have been reported for homoeologous pairs involving wheat chromosomes 2B, 3B, and 7B [32,64,65]. In contrast to homologous recombination in wheat, which occurs primarily in distal regions with a gradient from telomeres to centromeres correlated with gene density [66,67,68], the homoeologous pairs examined here exhibited a distinct meiotic recombination pattern.

Pyramiding resistance genes from diverse sources is a promising strategy for durable FHB control. The 1E-1B recombinant lines developed in this study, along with the physical mapping of the FHB resistance locus on chromosome 1E, provide valuable genetic resources and foundational information for pyramiding this novel resistance into elite wheat backgrounds. The integrated genomic-cytogenetic strategy demonstrated here offers an efficient framework for targeted introgression of wild relative genes into wheat, supporting ongoing efforts to enhance FHB resistance through precision breeding.

## 4. Materials and Methods Plant Materials

### 4.1. Plant Materials

The CS *ph1b* mutant were supplied by the wheat Genetics Resource Center at Kansas State University, USA. The CS-*Th. elongatum* disomic substitution lines DS 1E(1B) was originally provided by J. Dvorak at University of California, Davis, USA, and were maintained in Shanxi Agricultural University, China.

### 4.2. Population Development

DS 1E(1B) and CS *ph1b* mutant (*ph1bph1b*) were used to develop the special genotype that was double monosomic for *Th. elongatum* 1E and wheat chromosomes 1A or 1B and homozygous for *ph1b* allele as illustrated in Figure 1. In the Fall of 2022, we crossed the DS 1E(1B) line with the CS *ph1b* mutant. The resulting F_1_ hybrids were backcrossed to the CS *ph1b* parent in the Spring of 2023. All plants were grown in a greenhouse. BC_1_F_1_ seedlings were then genotyped using the *ph1b*-specific molecular markers Xwgc2111 and Xwgc2049 to select individuals homozygous for the *ph1b* allele [69]. The selected BC_1_F_1_ individuals were kept and were self-pollinated to induce homoeologous recombination between chromosome 1E with 1B. 1E-1B homoeologous recombinants were expected to be identified from the BC_1_F_2_ progenies.

### 4.3. Cytogenetic Analysis

Fluorescent genomic in situ hybridization (FGISH) was performed to differentiate *Th. elongatum* chromatin from wheat chromatin as described by Cai [70]. Total genomic DNA of *Th. elongatum* was used as GISH probes and was labeled with DIG-High Prime DNA Labeling and Detection Starter Kit I (Roche, REF: 11277065910). The sequence of Oligo-p119.2-1 and Oligo-pTa535-1 were obtained from Tang et al. [71]. The probe of Oligo-p119.2-1 and Oligo-pTa535-1 were synthesized and labeled with Biotin and Digoxigenin, respectively (Sangon Biotech Co., Ltd., Shanghai, China). Total CS genomic DNA was used as blocking after being sheered in boiling 0.4 M NaOH for 40–50 min. The *Th. elongatum* E genome probe and oligo pSc119.2-1 applied simultaneously under the hybridization condition of 69 °C for 2 min. Hybridization signals of Oligo-p119.2-1 was detected with fluorescein isothiocyanate-conjugated avidin (FITC-avidin) (Vector Laboratories, Inc., Newark, CA, USA) as green. Hybridization signals on *Th. elongatum* chromatin and Oligo-pTa535-1 were detected with Anti-Digoxigenin-Rhodamine (Roche, REF: 11207750910) as red, and wheat chromatin was counter-stained with DAPI (Roche, REF**:** 10236276001) as blue. Genomic in situ hybridization signals and chromosomes were visualized and analyzed under a fluorescence microscope (BX73, Olympus, Bartlett, TN, USA).

### 4.4. KASP Assay Analysis

The KASP assay genotyping was conducted following the protocol described by He [72]. The reaction of KASP assay was performed in 10 µL reaction volumes consisting of 5 µL of HiGeno 2 × Probe Mix B (Beijing JasonGen Biological Technology Co., Ltd., Beijing, China), 1.4 µL of primer mix, 2.6 µL of ddH_2_O, and 1.0 µL of template DNA (100 ng/µL). The PCR program was 94 °C for 5 min; 10 cycles of 94 °C for 20 s and 63–57 °C (each cycle decreased 0.5 °C and each temperature was kept for 30 s); then 30 cycles of 94 °C for 20 s and 58 °C for 1 min. KASP assays were performed using the Bio-Rad CFX real-time PCR system and Bio-Rad CFX Manager 3.1 software (Bio-Rad, Hercules, CA, USA).

### 4.5. Wheat 130K SNP Array and Bioinformatics Analysis

The identified 1E-1B recombinants, as well as the parental lines were genotyped by Wheat-130K SNP array from TCUni Biotechnology Co., Ltd. (Chengdu, China). The DNA extraction, library construction, probe capture and sequencing were performed following the manufacturer’s protocols. A multi-step bioinformatics pipeline was implemented for sequencing data processing. Initially, raw sequencing reads were subjected to quality control using fastp (v0.19.5) to eliminate low-quality sequences and adapter contamination [73]. Subsequently, the quality-filtered reads were aligned to the reference genome IWGSC RefSeq v2.1 using the BWA v0.7.16 mem algorithm [74]. The resulting alignment files (BAM format) were processed with Samtools v1.9 for sorting, PCR duplicate removal, and alignment statistics calculation [75]. Variant calling was performed following the GATK v4 best practices workflow [76], sequentially employing BaseRecalibrator, ApplyBQSR, HaplotypeCaller, CombineGVCFs, and GenotypeGVCFs modules, utilizing only high-quality aligned reads with minimum mapping quality ≥20. Finally, the identified variants were functionally annotated using a customized database developed with snpEff [77].

### 4.6. Physical Map Construction

Target SNPs derived from the wheat 130K SNP array, which had unambiguous physical positions on chromosome 1B, were selected to characterize the 1E-1B recombinants. Due to sequence homology between the two wheat and *Th. elongatum*, a considerable number of probes do hybridize to *Th. elongatum* loci. The captured sequences were sequenced and aligned to the Chinese Spring (CS) reference genome, with alleles coded as: reference allele “0”, alternative allele “1”, and NULL allele “–“. During data processing, reads with low sequencing depth or poor mapping quality were filtered out, which led to some *Th. elongatum* loci being assigned as NULL alleles (“–“). Based on these criteria, a physical map was constructed for each translocated chromosome, with breakpoints and fusion sites identified through SNP genotyping. The distribution of breakpoints was analyzed by scanning the physical map using 10 Mb sliding windows with 5-Mb steps on chromosome 1B.

### 4.7. FHB Disease Evaluation and Data Analysis

The 1E-1B recombinant lines and their parents were assessed for FHB resistance under greenhouse conditions via point inoculation, following the protocol of Zhu [78]. A single floret within a central spikelet was inoculated at anthesis by injecting 10 µL of a spore suspension. The inoculum was prepared by mixing equal spore numbers from four *Fusarium graminearum* isolates and adjusting the concentration to 1 × 10^5^ spores per mL. During the disease development period, plants were maintained in a greenhouse at approximately 25 °C with a 16 h photoperiod. To promote infection, high humidity was sustained for 72 h by covering the inoculated spikes with plastic bags. The disease evaluation included a minimum of six BC_1_F_3_ plants per genotype, distributed across three pots. At 21 days post-inoculation, FHB severity was recorded as the percentage of infected spikelets per spike, with a minimum of six spikes scored per genotype. An analysis of variance (ANOVA) was performed on the severity data, and means were separated using Fisher’s protected LSD test. All statistical analyses were carried out with R software (version 4.4.3) utilizing the “agricolae” package.

## Figures and Tables

**Table 1:** SNP-derived KASP markers for 1B-1E homoeologous recombinants screening.

Markers	SNP Alleles	SNP Chromosome Location	SNP Reference Location (bp) ^a^	Forward and Reverse Primers ^b^	Polymorphism
*EB1*	[G/A]	1B	1,518,466	F1:[Tail1]-5′CAGAGGTTCGAGGAAGCT 3′ F2:[Tail2]-5′ACCTGATGAGTCAAGAGTG 3′ R:5′CACTCCCTCGTAGAACGCG 3′	1B-1E
*EB2*	[A/G]	1B	383,971,145	F1:[Tail1]-5′GAGGAAGTGTTTCAGCTGTG 3′ F2:[Tail2]-5′GGAAGTCAAAAGGCGGC 3′ R:5′CATGCTTCAACTTCTTCCAGCTC3′	1B-1E
*EB3*	[A/T]	1B	697,751,963	F1:[Tail1]-5′CACAAAGTAATCATCCAGTGT 3′ F2:[Tail2]-5′CAACACAAAGTAATCCAGTGA3′ R:5′AGCCAAGCTGTATGGCTACAG 3′	1B-1E

^a^ SNP location in the IWGSC Reference Sequence v2.1 assembly (IWGSC RefSeq v2.1). ^b^ [Tail1] = GCAACAGGAACCAGCTATGAC; [Tail2] = GACGCAAGTGAGCAGTATGAC.

**Table 2:** FHB severity of CS, DS 1E(1B), and 1E–1B translocation lines.

Lines	Translocated Chromosomes	Mean FHB Severity (%) *	
		2024 Fall	2025 Spring
CS	1B	70.0 ± 6.4 ^b^	78.0 ± 7.5 ^a^
DS1E(1B)	1E	25.8 ± 5.8 ^d^	42.8 ± 6.5 ^bcde^
1E1B_354-6	1E·1E-1B	23.8 ± 6.2 ^d^	38.3 ± 6.3 ^de^
1E1B_322-1	1E·1E-1B	26.2 ± 6.7 ^d^	41.0 ± 3.0 ^cde^
1E1B_11-4	1E·1E-1B	29.3 ± 7.9 ^d^	36.0 ± 9.7 ^e^
1E1B_19-7	1E·1E-1B	47.3 ± 4.5 ^c^	52.3 ± 7.2 ^b^
1E1B_154-3	1E·1E-1B	47.0 ± 6.4 ^c^	46.8 ± 4.8 ^bcd^
1E1B_198-1	1E·1E-1B	48.0 ± 6.8 ^c^	51.5 ± 7.1 ^b^
1E1B_115-2	1E·1E-1B	50.0 ± 6.4 ^c^	53.5 ± 4.4 ^b^
1E1B_84-1	1E·1E-1B	51.0 ± 6.7 ^c^	50.0 ± 6.7 ^bc^
1E1B_96-5	1E·1E-1B	49.2 ± 7.7 ^c^	53.7 ± 5.5 ^b^
1E1B_257-1	1E·1E-1B	30.3 ± 6.6 ^d^	45.2 ± 5.6 ^bcde^
1E1B_125-3	1E·1E-1B	29.0 ± 5.3 ^d^	43.3 ± 5.6 ^bcde^
1E1B_347-5	1E·1E-1B	72.5 ± 8.7 ^b^	78.8 ± 16.6 ^a^
1E1B_204-9	1E·1E-1B	76.0 ± 12.7 ^ab^	73.3 ± 9.4 ^a^
1E1B_5-1	1E·1E-1B	72.3 ± 10.2 ^b^	77.5 ± 13.2 ^a^
1E1B_17-3	1B·1B-1E	83.2 ± 11.3 ^a^	75.8 ± 14.0 ^a^
1E1B_45-6	1B·1B-1E	71.0 ± 15.3 ^b^	79.3 ± 11.8 ^a^
1E1B_89-7	1B·1B-1E	72.5 ± 3.6 ^b^	75.5 ± 12.9 ^a^
1E1B_329-1	1B·1B-1E	83.7 ± 11.6 ^a^	82.7 ± 11.8 ^a^
1E1B_298-1	1B·1B-1E	76.0 ± 10.9 ^ab^	81.0 ± 6.4 ^a^

* FHB severity was recorded as the percentage of infected spikelets per spike, with a minimum of six spikes scored per genotype. Mean  ±  standard deviation, values followed by different letters are significantly different at α  =  0.05 level.

## Data Availability

The raw SNP array sequences are publicly available in the NCBI BioProject database under accession number PRJNA1356544. The newly developed translocation lines, along with any additional analyzed datasets, are available from the corresponding author upon reasonable request.

## Supplementary Materials

The following supporting information can be downloaded at: https://www.mdpi.com/article/10.3390/plants14243805/s1, Supplementary S1. The original allelic discrimination of KASP markers for each plant from the BC_1_F_2_ population. Supplementary S2. The 1E-1B recombinants and their chromosome constitutions. Supplementary S3. Selection of homozygous 1E-1B recombinants. Supplementary S4. General report of TCUni-Wheat 130K SNP array analysis. Supplementary S5. Composite bin map of chromosome 1B based on 130K SNPs genotyping of 1E-1B recombinants.
